# Probing the Hepatic Progenitor Cell in Human Hepatocellular Carcinoma

**DOI:** 10.1155/2013/145253

**Published:** 2013-02-26

**Authors:** Shu-Qin Jia, Jian-Jun Ren, Pei-De Dong, Xing-Kai Meng

**Affiliations:** ^1^Surgery Laboratory, The Affiliated Hospital, Inner Mongolia Medical University, Hohhot, Inner Mongolia 010050, China; ^2^Department of Surgery, The Affiliated Hospital, Inner Mongolia Medical University, Hohhot, Inner Mongolia 010050, China

## Abstract

*Objective*. The intrahepatic stem cells, also known as hepatic progenitor cells (HPCs), are able to differentiate into hepatocytes and bile duct epithelia. By exposure of different injuries and different hepatocarcinogenic regimens, the mature hepatocytes can no longer effectively regenerate; stem cells are involved in the pathogenesis of hepatocellular carcinoma. *Methods*. Immunohistochemistry was performed on 107 paraffin-embedded hepatocellular carcinoma specimens with the marker of hepatocyte and hepatocellular carcinoma (HepPar1), biliary differentiation (CK7,CK19), haemopoietic stem cell (HSC) (c-kit/CD117, CD34, and Thy-1/CD90), HPC specific markers (OV-6), and Ki-67, p53 protein. *Results*. HPCs can be identified in the tumor nodules, around the edge of tumor nodules, and in the portal tracts of the paracirrhosis nodules being positive in HepPar1, CK7, CK19, and OV-6, but they failed to immunostain with CD117, CD34, and CD90. The HPCs positive in Ki-67 are observed in the tumor and paracirrhosis tissues. In 107 specimens, 40.2% (43/107) HCC tissues expressed p53 protein, lower than that of the HPCs around the tumor nodules (46.7%, 50/107) and much higher than that of the HPCs around the paracirrhosis nodules (8.41%, 9/107). *Conclusion*. Human hepatocellular carcinogenesis may be based on transformation of HPCs, not HSCs, through the formation of the transitional cells (hepatocyte-like cells and bile ductal cells).

## 1. Introduction 

 In vitro, the cellular response of the liver to different injuries and different hepatocarcinogenic regimens involves cells at different levels in the liver lineage: the mature hepatocyte, the intrahepatic stem cell, and the haemopoietic stem cell (HSC) in the liver derived from circulating bone marrow stem cells [1, 2]. Each of these cell types may produce mature cells [3–5], but the mature hepatocytes play the main role in the liver regeneration. While during injury states in which the mature hepatocytes themselves can no longer effectively regenerate, stem cells are activated and may give rise to hepatocellular carcinomas by exposure of the hepatocarcinogen. However, the precise origin of the carcinoma is disputed. While it has been shown that hepatic stem cells play an important role in the regeneration of a severely damaged liver, the mechanisms by which they accomplish this are still as yet poorly understood.

 The intrahepatic stem cell, also known as hepatic progenitor cells (HPCs), are small epithelial cells with an oval nucleus and a small rim of eosinophilic cytoplasm that variably display features of both hepatocytes and bile duct epithelium. They are multipotential liver stem cells that are able to differentiate into hepatocytes and bile duct epithelia [6–8], via the formation of intermediate hepatocyte-like cell and bile ductular cells, respectively. Compared with “oval cells” and their progeny in animal models, many researchers have found that human HPCs have three subtypes with similar ultrastructural characteristics: the most undifferentiated cell, situated at the sinusoidal pole of hepatocytes; hepatocyte-like cell, located in hepatic cords; and bile ductal cell, exists in the portal tract [9–13]. Immunohistochemically, the monoclonal antibody OV-6 is useful in identifying these cells [14].

More and more evidences support that HPCs may give rise to carcinomas. The bipotential ability of HPCs to differentiate into hepatocytes and bile duct epithelia is involved in the pathogenesis of hepatocellular carcinoma (HCC) in animal models [15, 16]. But the precise roles of HPCs played underlying the development of HCC in human are not well understood. The aims of this study are to determine whether HPCs and HSCs could be detected in the primary HCC and their differentiation status. 

## 2. Methods

### 2.1. Patients and Tissue Samples

A total of 107 paraffin-embedded HCC specimens were obtained from the Department of Surgery at the Affiliated Hospital of Inner Mongolia Medical University. None of the patients had received chemotherapy or radiation therapy preoperatively. Each tissue includes tumor and paracirrhosis tissue and is large enough to access the comparison of both immunostaining results.

### 2.2. Immunohistochemistry

Sections (4 *μ*m thick) were made from the neutral formalin fixed and paraffin-embedded biopsies. Immunohistochemistry was performed on these sections with mouse monoclonal antibodies: the marker of hepatocyte and HCC (HepPar1), the markers of biliary differentiation (CK7 and CK19), the markers of HSC (c-kit/CD117, CD34, and Thy-1/CD90), the HPC specific markers (OV-6), Ki-67, and p53 protein. The data are summarized in Table 1. Primary antibodies were all incubated in the proper dilution with the sections overnight at 4°C and then were detected using the Powervision two-step histostaining reagent, with PV-6001, (Dako, Glostrup, Denmark) as the secondary antibody. The positive immunohistochemistry controls were routinely used.

 After staining, the sections were examined by two observers independently, and staining was recorded as positive if any strong staining was present, as was the cellular compartment in which this staining was present. 

## 3. Results

### 3.1. The Immunostaining of Stem Cells

**Figure 1:**
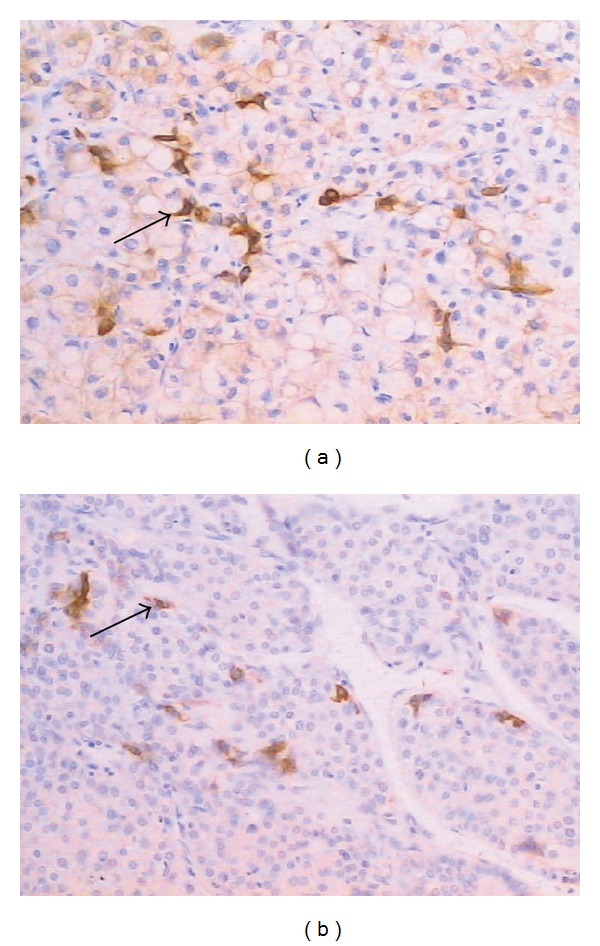
Hepatocyte-like cells scattered in the tumor nodules being positive in HepPar1 (a) and OV-6 (b) (×200 staining indicated by arrows). Cell nuclei were stained with hematoxylin (blue).

 Hepatocyte-like cell: The intermediate hepatocyte-like cells are polygonal cells with size and phenotype intermediate between progenitor cells and hepatocytes. They were scattered in the tumor nodules with a larger density that varied from area to area without a specific predilection for areas. But they were scarcely seen in the paracirrhosis tissue. Hepatocyte-like cells show the immunoreactivity for HepPar1 and OV-6 (Figure 1).

#### 3.1.1. Bile Ductal Cell

**Figure 2:**
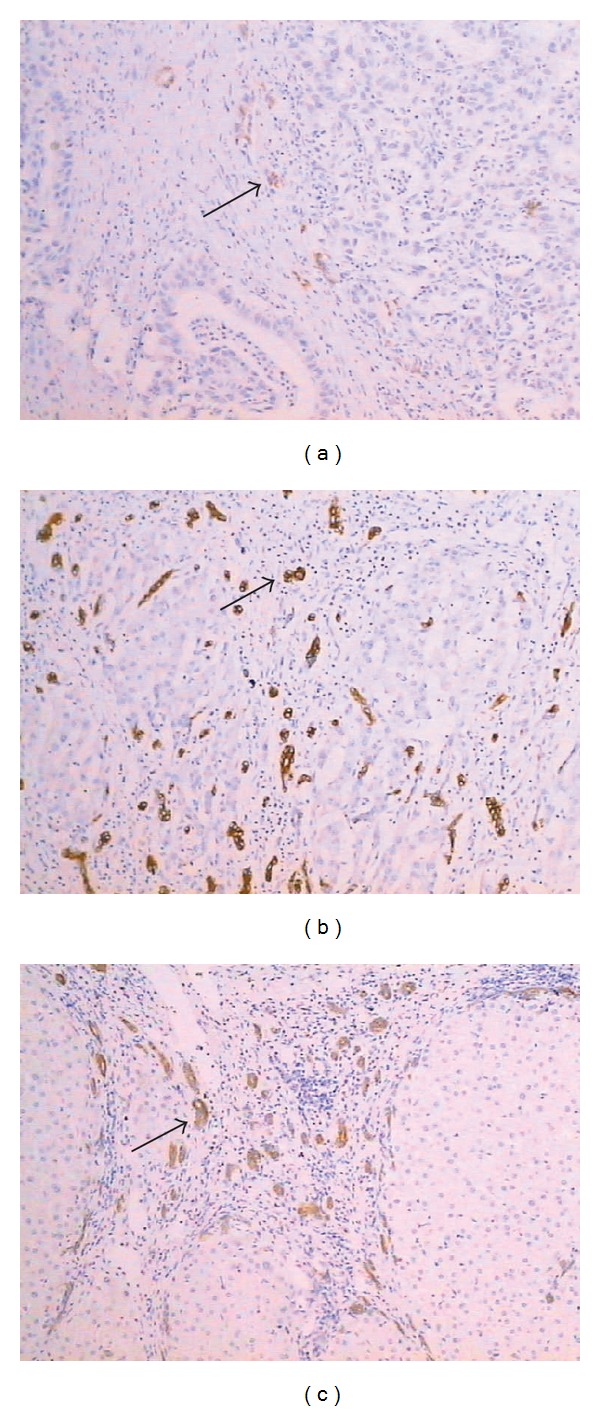
Bile ductular cells around the edge of tumor nodules were positive with CK7 (a), CK19 (b), and OV-6 (c) (×100 staining indicated by arrows). Cell nuclei were stained with hematoxylin (blue).

Light microscopy revealed that there exist many proliferating tubular or bilayered structures without obvious lumens lined by duct-like cells that have a higher nuclear/cytoplasmic ratio than normal duct cells in the portal tracts around the edge of tumor nodules and the paracirrhosis nodules, exactly tightly around the edge of tumor nodules. These bile ductular cells were positive with CK7, CK19, and OV-6 (Figure 2). 

#### 3.1.2. HSC

The markers of HSC, including CD117, CD34 (only positive in sinusoidal and vascular endothelium), and CD90, failed to show stem cells in the HCC tissues. The pictures are omitted. 

In all, HPCs exist in the area around the liver nodules and scatter in the nodules and are closely associated with the tumor cells. Interestingly, HPCs around the tumor nodules are more than those around the paracirrhosis nodules. The profile of markers' expression of HPCs is summarized in Table 2.

### 3.2. The Immunostaining of Tumor and Paracirrhosis Tissue

Tumor cells and the paracirrhosis hepatocytes are positive for HepPar1. In the paracirrhosis tissues, the “normal” hepatocytes also show the immunoreactivity for HepPar1. But the tumor cells and the “normal” hepatocytes stain weaker than the HPCs.

### 3.3. The Immunostaining of Ki-67 and p53 Protein

**Figure 3:**
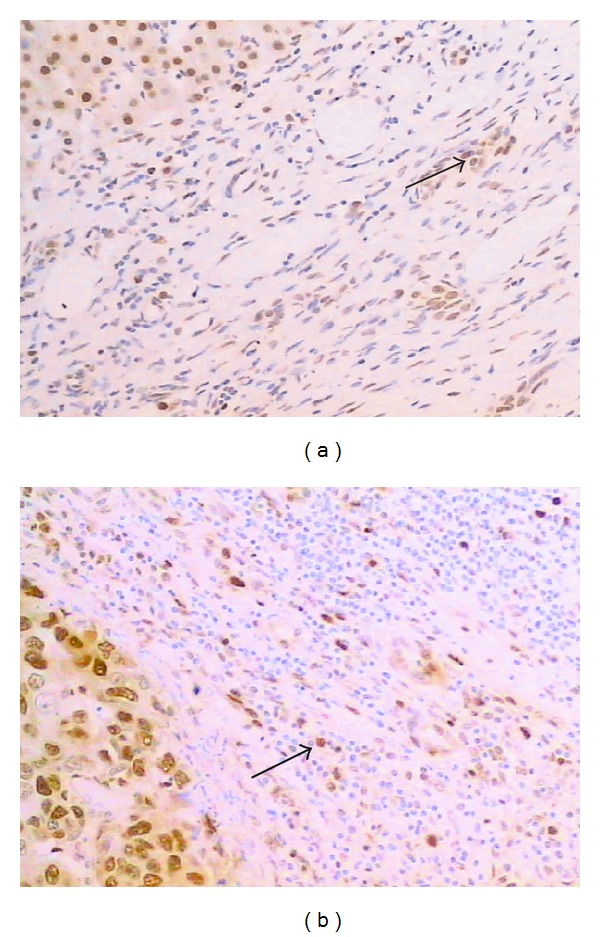
The HPCs around the tumor nodules expressed Ki-67 (a) and p53 protein (b) (×200 staining indicated by arrows). Cell nuclei were stained with hematoxylin (blue).

The slides were distinguished as negative and positive when the count of positive cells was less than 10% and over 10% for Ki-67 and p53 protein, respectively. The HPCs positive of Ki-67 are observed in the tumor (Figure 3(a)) and paracirrhosis tissues.

In 107 specimens, 40.2% (43/107) HCC tissues expressed p53 protein, lower than that of the HPCs around the tumor nodules (46.7%, 50/107) and much higher than that of the HPCs around the paracirrhosis nodules (8.41%, 9/107).

## 4. Discussion

In the human livers, there exist two types of stem cells: the haemopoietic stem cell from bone marrow and the intrahepatic stem cell existing in the Canals of Hering (CoH) [3, 13, 17, 18]. The latter is also known as HPC. In the normal human livers, the normal stem cells are generally quiescent, spending most of their time in G0 phase [19], and are hard to be identified. Only when liver damage is severe enough that large numbers of hepatocytes are lost and others' proliferation is suppressed by exposure to hepatotoxins or carcinogens, stem cells are activated and increased in number in the portal tracts around the liver nodules. More and more evidence supports that HPCs were observed to be associated with the development of liver carcinoma in animal models [15, 20, 21] and in human [13, 22–26]. 

Hematopoiesis and hepatic development share common stages. Simultaneous with the appearance of hematopoiesis in the fetal liver, HSC can be detected in the fetal liver. And the concentration of stem cells will decline with the loss of hepatic hemopoietic ability. Although the liver loses its hematopoietic functions, hematopoiesis often returns in adult life in disease states, probably with the recurrence of the HSC. CD90 is a cell surface marker, used in conjunction with CD34 and CD117 to identify HSCs. Many experiments supported the idea that the presence of stem cells expresses the HSC markers in the liver of the pathogenic model [27–29]. The negative expressions of these markers in this study imply that HSC does not exist in the HCC tissue. And it may imply that HPCs, but not HSCs, represent a potential target cell population for hepatocarcinogens or represent the cell of origin for the tumor.

As the experiment show, the hepatocyte-like cells and bile ductal cells show the superiority in number in the tumor than in the paracirrhosis tissue. Both of the cells are the transitional cells of HPCs differentiation to specific cell lineages. We can make the hypothesis further that human hepatocellular carcinogenesis may be based on transformation of HPCs through the formation of the transitional cells: hepatocyte-like cells and bile ductal cells. Ki-67 antigen is the prototypic cell-cycle-related nuclear protein, expressed by proliferating cells in all phases of the active cell cycle (G1, S, G2, and M phases). The amount of Ki-67 positive cells could reflect the proliferating activity of cells. As is shown in this study, HPCs are activated in great number and showed the proliferating activity around the tumor nodules. It further verifies our hypothesis mentioned above. Researchers may suspect that HPCs are only a small population in the tumor tissue; they are not able to proliferate and give rise to tumor cells. But experiments supported the idea that such stem cells represent only a small fraction of a tumor, as they possess the capability to regenerate a tumor, and most cancer cells lack this regenerative capability [30, 31]. Recent study also showed that high expression levels of putative HPC biomarkers were confirmed as significant predictors for overall survival and/or relapse-free survival of HCC [29]. 

By definition, cells in a side population have distinguishing biological characteristics (e.g., they may exhibit stem-cell-like characteristics), but the exact nature of this distinction depends on the markers used in identifying the side population. 

Side population is one of the most popular aspects in research work of stem cells [32]. In flow cytometry, a side population is a subpopulation of cells that is distinct from the main population on the basis of the markers employed. Since the side population cells have human breast carcinoma protein (Bcrp) on its membrane, they could be sorted by flow cytometry for effusing Hoechst-33342. Side populations have been identified in cancer and may be the cells that efflux chemotherapy drugs, account for resistance of cancer to chemotherapy. There are so many similar characters between stem cells and side population [32]. This method was firstly used to isolate HSCs from the bone marrow. We have performed information retrieval work, and there is no successful case of isolation side population cells from paraffin-embedded specimens. The morphological observations might be strengthened by other nonimmunological approaches to support our idea in future studies.

More than 90% of HCCs are accompanied by the cirrhosis in China. In other words, cirrhosis is the pre-malignant lesion of HCC. In HCC, there is always p53 gene mutant, which leads to the abnormal growth of cells and ultimately the occurrence of cell conversion and carcinoma changes. Because of the short *T*_1/2_ of wide-type P53 (WT-P53) protein, p53 protein detected by immunohistochemistry is almost mutant-type p53 (MT-P53) protein. In this study, 40.2% (43/107) HCC tissues expressed MT-p53 protein, lower than that of the HPCs around the tumor nodules (46.7%, 50/107) and much higher than that in the paracirrhosis tissue and HPCs around the paracirrhosis nodules (<10%). Through the tendency of P53 expression in different cells, we could observe more mutations in the HPCs around the tumor nodules than that in the HPCs around the paracirrhosis nodules. So we can finally conclude that the carcinogenesis might be the progress of the malignant transformation of the normal HPC that has accumulated oncogenic insults over time.

## Figures and Tables

**Table 1:** Primary antibodies and their dilution ratio.

Antigen	Catalogue Number	Supplier	Dilution ratio	Cell distribution
CK7	ab82253	abcam	1 : 200	Cytoplasm/membrane
CK19	ZM-0074	Zymed	1 : 200	Cytoplasm/membrane
HepPar1	ZM-0131	Zymed	1 : 100	Cytoplasm
OV-6	MAB2020	R&D	1 : 100	Cytoplasm
CD117	MS-271-P0	NeoMarker	1 : 100	Cytoplasm
CD34	ab27448	abcam	1 : 100	Cytoplasm/membrane
CD90	MAB2067	R&D	1 : 100	Cytoplasm
Ki-67	ab16667	abcam	1 : 200	Nucleus
P53	ab2433	abcam	1 : 100	Nucleus

**Table 2:** Summary of immunohistochemical expression analysis.

Marker	Hepatic progenitor cell				
	HLC	BDC	Hepatocyte	BEC	Tumor cell
CK7	−	+	−	+	−
CK19	−	+	−	+	−
HepPar1	+	−	+	−	+
OV-6	+	+	−	+	−

BDC: bile ductular cell in the para-cirrhosis tissue, BEC: bile epithelial cell; heptocyte: the relative normal hepatocyte in the para-cirrhosis tissue; HLC: hepatocyte-like cell.
